# An Amplitude Analysis-Based Magnetoelastic Biosensing Method for Quantifying Blood Coagulation

**DOI:** 10.3390/bios15040219

**Published:** 2025-03-29

**Authors:** Xi Chen, Qiong Wang, Jinan Deng, Ning Hu, Yanjian Liao, Jun Yang

**Affiliations:** 1 Key Laboratory of Biorheological Science and Technology, Ministry of Education and Bioengineering College, Chongqing University, Chongqing 400044, China; 20151901014@cqu.edu.cn (X.C.); wangqiong@cqu.edu.cn (Q.W.); biojdeng@cqu.edu.cn (J.D.); huning@cqu.edu.cn (N.H.); 2 Department of Basic Medicine, Chongqing Medical and Pharmaceutical College, Chongqing 401331, China

**Keywords:** blood coagulation, magnetoelastic sensors, thromboelastography (TEG), viscosity measurement, clot strength

## Abstract

Blood coagulation tests are crucial in the clinical management of cardiovascular diseases and preoperative diagnostics. However, the widespread adoption of existing detection devices, such as thromboelastography (TEG) instruments, is hindered by their bulky size, prohibitive cost, and lengthy detection times. In contrast, magnetoelastic sensors, known for their low cost and rapid response, have garnered attention for their potential application in various coagulation tests. These sensors function by detecting resonant frequency shifts in response to changes in blood viscosity during coagulation. Nevertheless, the frequency-based detection approach necessitates continuous and precise frequency scanning, imposing stringent demands on equipment design, processing, and analytical techniques. In contrast, amplitude-based detection methods offer superior applicability in many sensing scenarios. This paper presents a comprehensive study on signal acquisition from magnetoelastic sensors. We elucidate the mathematical relationship between the resonant amplitude of the response signal and liquid viscosity, propose a quantitative viscosity measurement method based on the maximum amplitude of the signal, and construct a corresponding sensing device. The proposed method was validated using glycerol solutions, demonstrating a sensitivity of 13.83 V^−1^/Pa^0.5^s^0.5^Kg^0.5^m^−1.5^ and a detection limit of 0.0817 Pa^0.5^s^0.5^Kg^0.5^m^−1.5^. When applied to real-time monitoring of the coagulation process, the resulting coagulation curves and maximum amplitude (MA) parameters exhibited excellent consistency with standard TEG results (*R*^2^ values of 0.9552 and 0.9615, respectively). Additionally, other TEG parameters, such as R-time, K-time, and α-angle, were successfully obtained, effectively reflecting viscosity changes during blood coagulation.

## 1. Introduction

Coagulation is a vital regulatory mechanism within the human blood system. Dysfunction in this mechanism can lead to pathological bleeding or thrombosis, with severe consequences such as stroke, myocardial infarction, and hemophilia. In recent years, the incidence and mortality rates of cardiovascular diseases have continued to rise, posing a significant threat to human health [1]. As a necessary test item for the clinical diagnosis and treatment of cardiovascular diseases, coagulation function testing is of great importance. Additionally, coagulation testing holds significant value in perioperative management and preoperative diagnosis, offering vital insights on a patient’s coagulation status to reduce the risk of intraoperative bleeding [2].

Coagulation testing represents a fundamental approach for evaluating the complex functionality of the human coagulation and fibrinolytic systems, which involve intricate physiological processes. Extensive research has led to the development of various models and hypotheses, most notably the “cascade theory” [3]. Current studies indicate that the coagulation process involves multiple factors and both endogenous and exogenous reaction pathways [4]. The culmination of these cascade reactions results in the formation of cross-linked fibrin networks and platelet aggregation, transforming blood or plasma from a fluid state to an immobile gel-like structure.

Over time, coagulation testing has evolved to encompass a diverse range of test items. Conventional coagulation laboratory tests (CCTs), such as prothrombin time (PT), activated partial thromboplastin time (APTT), and fibrinogen assays, enable the assessment of specific pathways or factors within the coagulation cascade. Driven by the increasing demand for real-time clinical testing, viscoelastic-based point-of-care tests (VE POCTs) have emerged [5]. These include rotational thromboelastometry (ROTEM), thromboelastography (TEG), and Sonoclot coagulation analyzers, which comprehensively evaluate the overall function of the coagulation system by analyzing viscoelastic changes during coagulation.

Among these methods, TEG has emerged as the most representative technique for measuring blood viscoelasticity. By continuously monitoring the viscoelastic changes in a blood sample during coagulation and generating a dynamic curve through data processing, TEG can intuitively analyze the entire process of coagulation and fibrinolysis. It also allows for the analysis of various coagulation parameters, including coagulation factors, fibrinogen levels, and platelet function [6]. Compared to other conventional methods, TEG can reflect the interaction between coagulation cascade reactions and platelets, providing a more comprehensive assessment of coagulation function. This makes it valuable for diagnosing related diseases and for applications in perioperative coagulation function assessment, prognosis evaluation, therapeutic monitoring, and guiding component blood transfusions. However, TEG instruments have drawbacks such as large size, high cost, and long detection times. For clinical point-of-care testing, there is an urgent need to develop small, rapid blood coagulation testing devices.

Magnetoelastic sensors represent an emerging wireless resonant detection technology that utilizes magnetostrictive materials as core sensing elements [7]. Based on the magnetoelastic characteristics of the material, these sensors measure changes in vibration characteristics by detecting the mutual coupling between mechanical deformation and magnetization processes. Magnetoelastic sensors offer high sensitivity, low operational costs, and compatibility with microfabrication technologies, making them suitable for integration into microsystems. They are also amenable to surface modification techniques to achieve a wide range of sensing properties [8]. Unlike other resonant detection sensors, magnetoelastic sensors rely on wireless magnetic field coupling, eliminating the need for an active power supply to the sensitive elements. This enables long-term, non-destructive detection in enclosed spaces, positioning them as a promising technology for applications in industrial monitoring [9,10,11], environmental monitoring [12,13,14], food safety [15,16,17,18], medical diagnostics [19,20,21], and more.

In coagulation testing, magnetoelastic sensors have been explored for various applications, including clotting time (CT) [22], prothrombin time (PT) [23], activated clotting time (ACT) [24], thromboelastography (TEG), and erythrocyte sedimentation rate (ESR) [25]. Compared to traditional methods, these magnetoelastic techniques offer cost-effectiveness and require smaller sample sizes, making them more suitable for point-of-care testing devices. Existing magnetoelastic coagulation detection technologies primarily rely on frequency analysis combined with clot-based assays. This involves adding a coagulation factor activator to plasma samples to induce in vitro coagulation. By monitoring shifts in the resonance frequency of the magnetoelastic response signal, real-time viscosity changes during coagulation are detected and converted into test results. According to existing theories [26], the relationship between the shift in resonance frequency and viscosity changes can be described as:

(1) $$\Delta f=-\frac{\sqrt{\pi f_{0}}}{2\pi \rho h_{s}}{\left(\eta_{L}\rho_{L}\right)}^{1/2}$$

where ρ and $\rho_{L}$ represent the densities of the sensitive element material and the measured liquid, respectively; *η_L_* denotes the dynamic viscosity of the measured liquid; *h_s_* is the thickness of the sensitive element; and *f*_0_ is the resonance frequency of the response signal. This theoretical foundation enables the quantitative detection of liquid viscosity through analysis of the resonance frequency of the magnetoelastic response signal. However, in practice, frequency detection is indirect and complex, imposing stringent requirements on equipment and detection methods. This is not conducive to rapid and portable clinical detection. For accurate frequency detection, high demands are placed on both sensor design [27,28] and signal analysis methods [29].

Sensor devices that rely on detecting resonance signals based on physical vibrations or wave spectra, such as piezoelectric quartz crystal sensors and surface plasmon resonance sensors, exhibit concurrent variations in the amplitude of the response signal as the resonance frequency shifts. Compared to frequency detection, amplitude detection is more accurate, direct, and easier to implement in engineering applications. In scenarios where the measured variable varies within a narrow range, the frequency scanning process can be omitted, thereby enhancing detection speed. Consequently, it is feasible to analyze viscosity changes during coagulation by focusing on the sensor’s response amplitude rather than frequency.

However, current theoretical research has yet to provide a definitive mathematical description of the relationship between the response amplitude of magnetoelastic sensors and parameters such as viscosity—a crucial indicator in coagulation analysis. As a result, existing studies on magnetoelastic sensors based on amplitude analysis often rely on statistical methods [30,31], which may compromise accuracy. For applications requiring precise quantitative analysis, such as coagulation testing akin to thromboelastography, current amplitude analysis methods for magnetoelastic sensors struggle to yield satisfactory results. Therefore, it is imperative to delve deeper into the theoretical models of magnetoelastic sensors to establish a clear analytical relationship between changes in sensor response amplitude and parameters like viscosity. Based on this, a rapid and accurate amplitude-based detection method for magnetoelastic sensors can be developed, catering to analytical applications such as quantitative liquid viscosity measurements.

This study comprehensively analyzes the interactions among magnetic fields, mechanical vibrations, and flow fields involved in the magnetoelastic detection of liquid samples such as blood. We provide a mathematical description of how the resonant amplitude of the response signal varies with the viscosity of the liquid under test. Based on this, an accurate amplitude analysis method for magnetoelastic liquid viscosity detection is proposed. Using this method, a portable magnetoelastic sensing device has been developed for coagulation testing, and a comparative analysis with standard thromboelastography has been conducted. The magnetoelastic coagulation sensing method based on amplitude analysis can generate dynamic coagulation curves similar to standard thromboelastograms and accurately obtain the maximum amplitude (MA) parameter, enabling real-time quantitative detection of the coagulation process.

## 2. Materials and Methods

### 2.1. Mathematical Description of the Relationship Between the Magnetoelastic Resonant Amplitude and the Liquid Viscosity

The magnetoelastic sensor operates on the principle of mutual coupling between the mechanical deformation and magnetization processes of its sensitive element. It can convert changes in mechanical vibration characteristics, induced by the measured variable, into electrical signals for detection. Magnetoelastic sensors are capable of responding to a wide range of parameters, including mass loading, temperature, viscous damping, gas and liquid flow rates, and external stress. During actual measurement, it is often necessary to maintain constant multiple parameters of the sample to detect changes in the response signal caused by a single parameter or a few related parameters.

In coagulation testing, changes in sample viscosity are a key parameter reflecting the coagulation process, while other parameters such as temperature and solution composition, which affect magnetoelastic signals, are relatively stable and easy to control. Therefore, detecting viscosity changes during coagulation through the sensor’s response signal is currently the primary method in magnetoelastic coagulation tests. Most existing magnetoelastic sensing theories are based on mechanical vibration models, which characterize changes in the measured variable through the resonant frequency of the response signal. This approach leverages the characteristic that the vibration of the sensitive element and the response signal are at the same frequency. However, to establish a magnetoelastic sensing method based on amplitude, it is necessary to determine the quantitative relationship between the resonant amplitude of the response signal and the viscosity of the liquid being measured.

**Figure 1:**
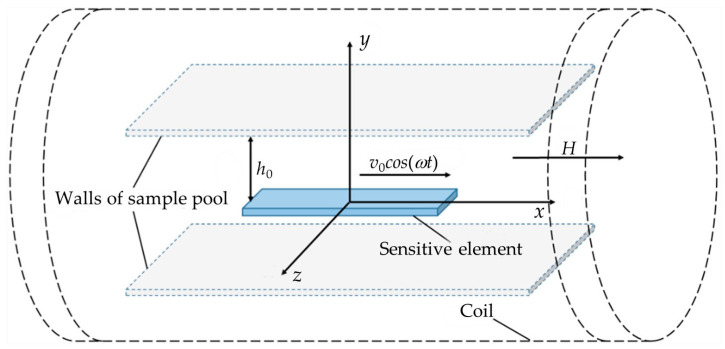
Schematic illustration of magnetoelastic liquid viscosity measurement.

In quantitative analysis, based on the existing magnetoelastic sensing frequency-domain description and the most common configuration of sensing devices [32,33], a continuous frequency scanning method is employed. Here, a solenoid coil is used both to generate the excitation magnetic field and to receive the response signal. The sensor’s sensitive element is placed unconstrained along the long axis of the coil within a closed or semi-closed sample cell containing the liquid to be measured. The sample cell is fixed at the center of the coil. As shown in Figure 1, the sample cell is positioned between the upper and lower walls, with the sensitive element of length *l_s_* resting statically in the pool, immersed to a depth of *h*_0_ in the liquid and surrounded by the solenoid coil outside the sample cell.

During sensor operation, an alternating current *I_AC_*sin(*ωt*) is continuously applied to the coil to generate the excitation magnetic field, which magnetizes the sensitive element, inducing magnetostrictive strain. Under the combined effects of viscous damping from the liquid environment and intrinsic damping, the magnetostrictive strain of the sensitive element exhibits steady-state harmonic vibrations at the same frequency as the excitation magnetic field. The velocity of the sensitive element’s motion is assumed to be *v*_0_cos(*ωt*), where *ω* is the excitation angular frequency. Due to the inverse magnetostrictive effect [34], the harmonic vibrations and deformation of the sensitive element cause corresponding changes in its magnetization state that ultimately induce an electromotive force, i.e., the response signal, in the coil. During detection, the frequency of the excitation signal is continuously varied, and resonance occurs when it approaches the natural frequency of the sensitive element, maximizing the amplitude of the response signal. Clearly, to determine the relationship between the resonant amplitude of the response signal and the viscosity of the measured liquid, it is necessary to clarify the influence of the excitation magnetic field, liquid viscous damping, and the sensor’s own damping on the vibration state of the sensitive element. These factors must then be correlated with the response signal.

The excitation magnetic field exerts its influence through two key processes: the magnetization of the sensitive element induced by the magnetic field, and the resulting magnetostrictive deformation. However, both processes exhibit strong nonlinear characteristics, complicating direct analysis. To address this complexity, we simplify these processes by referring to relevant research [35]. In magnetoelastic testing, the excitation magnetic field is typically small, while a large constant bias magnetic field is applied to enhance signal quality. As a result, the magnetization intensity and magnetostrictive strain of the sensitive element undergo small-scale dynamic variations superimposed on a constant state determined by the bias magnetic field. These variations can be approximated as linear. During the magnetization process, the change in magnetization intensity of the sensitive element exhibits a first-order linear relationship with the alternating current (AC) excitation magnetic field. The linear coefficient is defined as the AC susceptibility (*χ_AC_*), which corresponds to the slope of the magnetization curve of the sensitive element material at the position determined by the detection bias magnetic field. In the magnetostrictive process, the linear coefficient linking material deformation to changes in magnetization intensity is the magnetostrictive linear coefficient *d_λ_* = 3*λ_s_M_DC_*/*M_s_*^2^, where *λ_s_* and *M_s_* are the material’s saturation magnetostrictive coefficient and intensity, respectively, and *M_DC_* is the magnetization intensity under the bias magnetic field. These parameters and the excitation signal *I_AC_*sin(*ωt*) are all known during detection. Given the coil length *l_c_*, the number of turns *N*, and the magnetic leakage coefficient *k_m_*, the intensity of the excitation magnetic field can be approximately calculated using Ampere’s circuital law. By combining the above analyses of the magnetization and magnetostrictive processes, a direct relationship between the excitation signal and the magnetostrictive deformation of the material can be established, thereby clarifying the effect of the excitation magnetic field on the sensitive element.

However, incorporating the effects of the magnetic field directly into mechanical vibration models is challenging. Therefore, the deformation caused by the excitation magnetic field is equivalently treated as deformation induced by stress. Assuming that an alternating concentrated force *F*_0_sin(*ωt*) is applied to one end of the sensitive element instead of the excitation magnetic field, the mechanical deformation is identical to the magnetostrictive deformation produced by the excitation magnetic field. According to Hooke’s law, both the mechanical deformation and the magnetostrictive deformation can be calculated separately and are equal, denoted as *ε*:

(2) $$\varepsilon =\frac{F_{0}}{EA_{s}}\sin\omega t=d_{\lambda }\chi_{AC}\frac{NI_{AC}}{k_{m}l_{c}}\sin\omega t$$

where *E* represents the Young’s modulus of the material, corrected for the Δ*E* effect and Poisson’s ratio [36], and *A_s_* is the cross-sectional area of the sensitive element. The amplitude *F*_0_ of the equivalent force can be determined. By introducing the Dirac delta function *δ* to indicate the location of its application, the equivalent force *F* resulting from the excitation magnetic field can be expressed as:

(3) $$F(x,t){=F}_{0}\sin\left(\omega t\right)\delta (x-\frac{l_{s}}{2})=d_{\lambda }EA_{s}\chi_{AC}\frac{NI_{AC}}{k_{m}l_{c}}\sin\left(\omega t\right)\delta (x-\frac{l_{s}}{2})$$

During sensing and detection, all parameters in Equation (3) can be determined, allowing the effect of the excitation magnetic field on the sensitive element to be clearly identified through the equivalent force *F*.

The influence of liquid viscous damping on the vibrational state of the sensitive element primarily arises from the frictional damping force generated by the relative motion between the measured liquid and the sensitive element. During calculation and analysis, the measured liquid can be simplified as an ideal Newtonian fluid. According to Newton’s law of internal friction [37], the liquid damping force is the product of the liquid’s viscosity and velocity. Here, the viscosity is the variable quantity being measured, while the velocity needs to be determined based on the motion state. The sensitive element undergoes harmonic vibration under the action of the magnetic field, driving the measured liquid to perform periodic motion. In Figure 1, the spatial structure of the sample cell is regular, allowing the approximation that the liquid only moves as laminar flow in planes parallel to the *xOz* plane and propagates and attenuates as transverse waves along the *y*-axis. In magnetoelastic coagulation detection, the excitation signal frequency is typically above several hundred kHz, and the viscosity of the blood being measured is relatively low. Therefore, the propagation of liquid motion attenuates rapidly, with a propagation distance usually in the micrometer range, far less than the size of the sample cell. The sample cell can be approximated as an infinite space. Referring to Stokes’ second problem [37], for a liquid with density $\rho_{L}$ and viscosity *η_L_*, its velocity *v_L_* only depends on the position along the *y*-axis and time. This allows for the derivation of simplified Navier–Stokes equations and corresponding boundary conditions:

(4) $$\left\{\begin{array}{l} \;\;\;\;\rho_{L}\frac{\partial v_{L}}{\partial t}=\eta_{L}\frac{\partial^{2}v_{L}}{\partial t^{2}}\\ \;\;v_{L}\left(0,t\right)=v_{0}\cos\omega t\\ v_{L}\left(\infty ,t\right)=0 \end{array}\right.$$

By solving Equation (4), we can determine the motion state of the liquid and, subsequently, the relative motion velocity of the liquid on the surface of the sensing element. As a result, the shear friction force *F_τ_* due to the liquid motion is given by:

(5) $$F_{\tau }=\eta_{L}{\left.\frac{\partial v_{L}\left(y,t\right)}{\partial y}\right|}_{y=0}=-\sqrt{\frac{\omega \eta_{L}\rho_{L}}{2}}v_{0}\cos\omega t$$

It is important to note that, at any given moment, the vibration velocity and the shear friction force experienced by different parts of the sensing element are not uniform. Therefore, we analyze a microsegment *dx* along the long axis of the element. Assuming that the displacement of the microsegment located at a distance *x* from the center of the sensing element at any time is *u*(*x*,*t*), the velocity of this microsegment can be expressed as the partial derivative of the displacement with respect to time, i.e., *v*_0_cos*ωt* = *∂u*(*x*,*t*)/*∂t*. The total damping force exerted on this microsegment by the liquid from its upper and lower surfaces can be represented as:

(6) $$F_{\eta }=2F_{\tau }w_{s}dx=-w_{s}\sqrt{2\omega \eta_{L}\rho_{L}}\frac{\partial u\left(x,t\right)}{\partial t}dx$$

In addition to the viscous damping effect of the liquid, the inherent damping characteristics must also be considered when determining the vibrational state of the sensing element. The sensing element is made of ferromagnetic material, and the damping it experiences during detection is relatively complex. In addition to mechanical damping, other elastic energy losses, such as eddy current loss and elastic hysteresis [34], are present. Each of these mechanisms has distinct influencing factors. However, during the detection process, these damping effects are largely constant and independent of the liquid. Therefore, they can be simplified as the sensor’s inherent mechanical viscous damping, characterized by a linear damping constant *D*_0_.

After characterizing the excitation magnetic field, liquid viscous damping, and the sensor’s inherent damping, the vibrational state of the sensing element is analyzed using elastodynamics [38]. By analyzing a microsegment along the long axis of the sensing element, it can be deduced that, during vibration, the resultant force acting on the microsegment, including the equivalent force from the excitation magnetic field, the liquid damping force, the sensor’s damping force, and the elastic tensile forces at both ends, must balance with the inertial force of the microsegment’s motion. Combining Equations (3) and (6), the vibration equation of the sensing element during the sensor’s detection process can be expressed as:

(7) $$A_{s}\rho \frac{\partial^{2}u\left(x,t\right)}{\partial t^{2}}+\left(D_{0}+w_{s}\sqrt{2\omega \eta_{L}\rho_{L}}\right)\frac{\partial u\left(x,t\right)}{\partial t}-A_{s}E\frac{\partial^{2}u\left(x,t\right)}{\partial^{2}x}=F(x,t)$$

where *w_s_* represents the width of the sensing element, ρ denotes its density, and *ω* is the angular frequency of the excitation magnetic field, which is also the angular frequency of the excitation electrical signal. Solving this equation allows us to obtain the vibration displacement of the sensing element. We define $\beta_{L}=\left(D_{0}+w_{s}\sqrt{2\omega \eta_{L}\rho_{L}}\right)/2A_{s}\rho$. Consequently, the steady-state solution for the vibration displacement of the sensing element can be expressed as:

(8) $$\begin{array}{l} u\left(x,t\right)=-\frac{4d_{\lambda }E\chi_{AC}NI_{AC}}{k_{m}l_{c}\rho l_{s}}\frac{1}{\sqrt{{\left(\omega^{2}-\omega_{0}^{2}\right)}^{2}+4{\beta_{L}}^{2}\omega^{2}}}\sin\left(\frac{\pi }{l_{s}}x\right)\cos\left(\omega t-\theta_{u}\right)\\ \;\;\;\omega_{0}=\frac{\pi }{l_{s}}\sqrt{\frac{E}{\rho }}\\ \;\;\;\theta_{u}=\tan^{-1}\left(\frac{\omega^{2}-\omega_{0}^{2}}{2\beta_{L}\omega }\right) \end{array}$$

where *ω*_0_ represents the intrinsic frequency of the sensing element, and *θ_u_* denotes the phase difference between the mechanical vibration of the sensing element and the excitation signal. This equation describes the vibration state of the sensing element during magnetoelastic testing and establishes a relationship between the vibration state and the viscosity of the liquid.

After determining the vibrational state of the sensing element, it is correlated with the sensor’s response signal to establish the relationship between the resonant amplitude of the response signal and the viscosity of the liquid. The deformation is calculated from the vibrational state, the magnetization is derived from the deformation, and ultimately, the response signal is computed based on the changes in the magnetic field. The total deformation of the sensing element is obtained by integrating the displacement solution in Equation (8) over the length of the element. Furthermore, based on the magnetostrictive constitutive equation [39], the corresponding magnetization state is derived from the deformation of the element:

(9) $$\begin{array}{c} M\left(\varepsilon \right)\&=\left(\frac{\partial M}{\partial \sigma }\right)\left(\frac{\partial \sigma }{\partial \varepsilon }\right)\varepsilon \left(t\right)=\frac{1}{\mu_{0}}\left(\frac{\partial \varepsilon }{\partial M}\right){\left(\frac{\partial M}{\partial H}\right)}_{\sigma }\left(\frac{\partial \sigma }{\partial \varepsilon }\right)\varepsilon \left(t\right)\\ \;\;\;\;\;=\frac{1}{\mu_{0}}d_{\lambda }\chi_{AC}E\frac{2}{l_{s}}\left[u\left(\frac{l_{s}}{2},t\right)-u\left(0,t\right)\right] \end{array}$$

where *μ*_0_ represents the magnetic permeability of vacuum. The calculation of the response voltage involves summing the two magnetic fluxes generated within the coil by the magnetization of the sensing element and the excitation magnetic field, and then taking the derivative. Assuming the cross-sectional area of the coil is *A_c_*, the response voltage of the sensor can be expressed as:

(10) $$U\left(t\right)=\frac{N^{2}I_{AC}}{k_{m}l_{c}}\omega \left[A_{c}\mu_{0}\cos\left(\omega t\right)+\frac{8A_{s}}{\rho }{\left(\frac{d_{\lambda }E\chi_{AC}}{l_{s}}\right)}^{2}\frac{\sin\left(\omega t-\theta_{u}\right)}{\sqrt{{\left(\omega^{2}-\omega_{0}^{2}\right)}^{2}+4\beta_{L}^{2}\omega^{2}}}\right]$$

The response signal already incorporates the influence of the viscosity of the measured liquid, but its mathematical form remains relatively complex. Analysis of Equation (10) reveals that the response is a sinusoidal periodic signal with the same frequency as the excitation signal but with a phase difference. It consists of two parts: the pure excitation magnetic field response and the response from the sensing element. The excitation magnetic field response is not correlated with the measured quantity and can be predetermined and subtracted from the detection results. In detection applications, the change in the amplitude of the response signal is usually reflected by measuring the effective value to determine the resonance point. Assuming that the signal processing can completely remove the response corresponding to the excitation magnetic field, the amplitude of the response signal corresponding to the vibration of the sensing element can be extracted as:

(11) $$U_{id}=\frac{8A_{s}}{\rho }\frac{N^{2}I_{AC}\omega }{k_{m}l_{c}}{\left(\frac{d_{\lambda }E\chi_{AC}}{l_{s}}\right)}^{2}\left[\frac{1}{\sqrt{{\left(\omega^{2}-\omega_{0}^{2}\right)}^{2}+4\beta_{L}^{2}\omega^{2}}}\right]$$

This equation describes the response signal amplitude as a function of the excitation frequency *ω*, with its functional curve approximating an inverted bell-shaped. The maximum value point corresponds to the resonance point of the response. Analysis of this formula reveals that the resonant frequency corresponding to the maximum value is given by $\omega_{R}=\sqrt{\omega_{0}^{2}-2\beta_{L}^{2}}$, and the corresponding resonance voltage amplitude is:

(12) $$U_{R}=\frac{8A_{s}}{\rho }\frac{N^{2}I_{AC}\omega }{k_{m}l_{c}}{\left(\frac{d_{\lambda }E\chi_{AC}}{l_{s}}\right)}^{2}\left[\frac{1}{2\beta_{L}\sqrt{\omega_{0}^{2}-\beta_{L}^{2}}}\right]$$

Given that the damping coefficient *β_L_* is typically much smaller than the natural frequency *ω*_0_, the quadratic term of the damping coefficient can be neglected, leading to $\sqrt{\omega_{0}^{2}-\beta_{L}^{2}}\approx \omega_{0}$. Additionally, the damping coefficient *β_L_* is related to the excitation signal frequency *ω* and may vary during the frequency sweep detection process. However, since this variation is much smaller than its own value, it can be approximated by substituting the natural frequency *ω*_0_. By treating the square root of the product of the liquid’s viscosity and density $\sqrt{\eta_{L}\rho_{L}}$ as a single variable and rearranging Equation (12), we obtain:

(13) $$\frac{1}{U_{R}}=\left[\frac{w_{s}\sqrt{2\omega_{0}}k_{m}l_{c}}{8{A_{s}}^{2}N^{2}I_{AC}}{\left(\frac{l_{s}}{d_{\lambda }E\chi_{AC}}\right)}^{2}\right]\sqrt{\eta_{L}\rho_{L}}+\frac{{D_{0}k}_{m}l_{c}}{8{A_{s}}^{2}N^{2}I_{AC}}{\left(\frac{l_{s}}{d_{\lambda }E\chi_{AC}}\right)}^{2}$$

In this equation, all terms except the measured variable $\sqrt{\eta_{L}\rho_{L}}$ are constants. Its mathematical structure indicates a linear relationship between the reciprocal of the voltage amplitude at the resonance point of the response signal and $\sqrt{\eta_{L}\rho_{L}}$. This measured variable $\sqrt{\eta_{L}\rho_{L}}$ characterizes the effect of liquid viscous damping on the response signal and may be conveniently defined as the viscous damping value. According to Equation (13), the viscous damping value of the liquid can be obtained by analyzing the amplitude of the magnetoelastic sensor response signal. For liquids with known densities, this method can be used to calculate viscosity, and vice versa.

### 2.2. Magnetoelastic Measurement Device and Sensitive Element Fabrication

**Figure 2:**
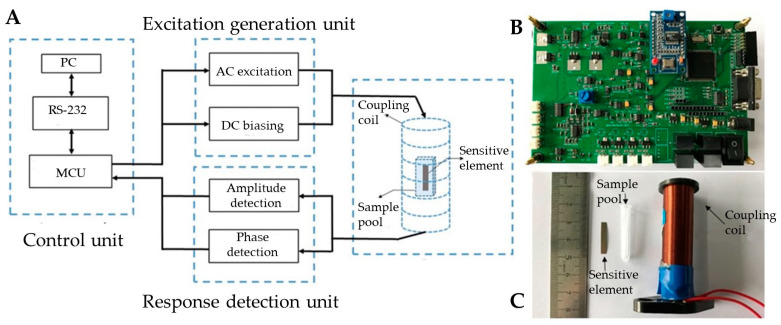
Magnetoelastic measurement device. (**A**) Block diagram of the device; (**B**) fabricated circuit board; (**C**) sensitive element, sample pool, and solenoid coil (serving as the coupling coil). During measurement, the sensitive element is positioned within the sample pool, which is secured at the center of the coil.

Building upon the magnetoelastic sensing method with amplitude analysis described earlier, this paper presents the development of a sensing and detection device, as illustrated in Figure 2A. Specifically, the excitation generation unit employs a direct digital synthesizer (DDS) and a digital-to-analog converter (DAC) to generate an adjustable-frequency AC excitation signal and a DC bias signal, respectively. The excitation generating unit produces excitation signals to vibrate the sensor element and bias signals to stabilize the sensor’s operation. After proper conditioning and power gain, they are converted into current and directly input into the coupling coil, generating response voltage signals on the coil. After high-pass filtering, the response voltage signals are fed into the response detection unit to prevent bias signals from interfering with amplitude detection. The response detection unit utilizes a root mean square (RMS) to DC converter and an analog-to-digital converter (ADC) to measure the resonant amplitude of the response signal. Additionally, a square wave duty cycle conversion method is implemented to measure the phase difference, thereby facilitating the accurate determination of resonance points. The control unit, comprising a microcontroller, orchestrates the operation of the aforementioned components and communicates with the host computer via a serial interface. These three units collectively form the primary components of the device’s circuitry, as depicted in Figure 2B. The coupling unit, as shown in Figure 2C, is rigidly assembled from a coil, a sample cell, and a sensing element. The solenoid coil features a length of 40 mm, an inner diameter of 12 mm, and is wound with 500 turns of 0.08 mm enameled copper wire in a single layer. The sample cell is a disposable plastic vial with dimensions of 30 mm in length and 5 mm in diameter.

The sensitive element is selected as AN101 Fe-based amorphous alloy ribbon, sourced from Advanced Technology & Materials (Beijing, China). This material demonstrates comparable performance characteristics to the widely used Metglas 2826MB (Conway, SC, USA). The ribbon is precisely cut into thin flakes with dimensions of 18 mm in length, 3 mm in width, and 29 µm in thickness using UV laser cutting technology. Subsequently, the cut sensitive elements are subjected to ultrasonic cleaning in anhydrous ethanol for 20 min and then dried using nitrogen gas.

### 2.3. Chemicals and Apparatus

Glycerol was purchased from Sinopharm Chemical Reagent (Shanghai, China). Quality control samples, simulated plasma samples, and activators were obtained from Dingrun Medical Equipment (Chongqing, China). The quality control and simulated plasma samples were derived from porcine plasma products, while the activator consisted of a solution containing kaolin and calcium chloride. The TEG 5000 hemostasis analyzer was sourced from Haemonetics (Boston, MA, USA).

### 2.4. Measurements of Glycerol Solutions by Amplitude Analysis

Based on the linear relationship between the resonant amplitude of the response signal from the magnetoelastic sensor and the viscosity and density of the liquid, liquid viscosity can be determined through amplitude analysis. For this study, glycerol solutions with concentrations ranging from 0% to 80% (in mass percentage) were prepared by mixing glycerol with triple-distilled water at 10% concentration intervals. These solutions were preheated in a constant-temperature water bath maintained at 20 °C, while the ambient experimental temperature was also kept at 20 °C. During the measurement, the sensing device was configured with an excitation current of 0.05 mA and a bias current of 3 mA. The procedure involved transferring 300 µL of the glycerol solution to be tested into a sample cell containing a sensitive element. The device then initiated a frequency sweep from 110 kHz to 135 kHz, with a step interval of 50 Hz, and recorded the response signals to complete the measurement. After each measurement, the sensitive element was removed, rinsed with distilled water, dried, and reused for subsequent tests. Each glycerol solution, as well as distilled water, was measured 20 times following the described procedure to ensure data reliability and repeatability.

### 2.5. Monitoring the Blood Coagulation

To verify the feasibility of amplitude analysis as a sensing method for coagulation monitoring, TEG quality control samples and simulated plasma samples were used in conjunction with an activator to trigger the endogenous coagulation pathway, thereby mimicking the coagulation response process. All samples and reagents were preheated in a constant-temperature water bath at 37 °C prior to experimentation. Additionally, due to the difficulty in cleaning the sensor’s sensitive element after the coagulation reaction, the sensitive element was replaced after each measurement to ensure accuracy. Given the inherent variability among the processed sensitive elements, each element used in the experiments required pre-recording of its response curve in air. This step facilitated subsequent data processing and minimized potential errors.

Quality control samples, which serve as calibration standards for the TEG analyzer, accurately simulate specific coagulation processes and particular clot firmness (corresponding to the MA value parameter on the TEG analyzer). Initially, six quality control samples were selected to establish a relationship curve between the response amplitude of the magnetoelastic sensor and blood clot firmness. The clot firmness of these samples, equivalent to the viscosity after reaction, increases in a gradient within the measurable range of the TEG analyzer. The magnetoelastic detection process involved adding 280 µL of the sample to the sample chamber, inserting the sensitive element, adding 20 µL of the activated coagulation reagent, and initiating the frequency sweep detection. The device settings were identical to those used for glycerol measurements, with continuous frequency sweep detection lasting for 20 min and real-time recording of response data. Concurrently, the same samples were tested using a TEG 5000 hemostasis analyzer, operated according to the manufacturer’s instructions. Each quality control sample underwent five replicate experiments following this procedure.

Subsequently, eight simulated plasma samples were randomly selected to further validate the method. These samples were designed to mimic various normal and abnormal human plasma conditions. The operational procedures were identical to those used for the quality control samples, with both the magnetoelastic device and the TEG analyzer employed simultaneously for comparative measurements on the same sample.

## 3. Results and Discussion

### 3.1. Verification of Amplitude Analysis Method Based on Glycerol Samples

The magnetoelastic viscosity measurement method based on amplitude analysis fundamentally relies on the linear relationship between the reciprocal of the sensor’s resonant amplitude (as described by Equation (13)) and the square root of the product of the density and viscosity of the measured liquid $\left(\sqrt{\eta_{L}\rho_{L}}\right)$. However, due to the introduction of approximations and simplifications in the analysis process, it is essential to verify the accuracy of the model. To this end, glycerol solutions with controllable viscosity were selected as standard samples for measurement, and the experimental results were compared and analyzed with numerical calculations.

In magnetoelastic measurements, several steps are taken to minimize interference from the excitation magnetic field and coil noise. Firstly, a pre-measured response of an empty coil is uniformly subtracted from the measured response signal during processing. This step effectively eliminates background noise and ensures accurate signal detection. Additionally, to facilitate comparative analysis, the signal gain and attenuation in the sensing device’s circuit are removed, and the effective value of the response is restored to its original amplitude. Furthermore, a low-pass digital filter is applied to the frequency-sweep response curve to remove high-frequency noise, thereby enhancing the clarity of the resonance point identification.

**Figure 3:**
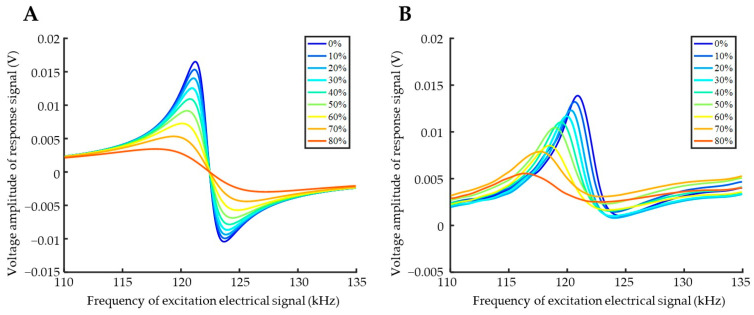
Frequency scanning response of glycerol solution measurement. (**A**) Numerical simulation results; (**B**) experimental measurement results.

The magnetoelastic sensing detection results (sensing response curves) of glycerol solutions with varying concentrations, obtained using the experimental setup, are presented in Figure 3B. These results are compared and analyzed with the numerical calculation results derived from Equation (10) (Figure 3A). In general, as the viscosity of the tested liquid increases, both the experimental and calculated results demonstrate a decrease in the frequency and amplitude corresponding to the resonance peak in the response curve. This trend indicates a broad consistency between the two sets of data. However, discrepancies between the calculated and experimental results are evident in the more pronounced anti-resonance peaks observed in the calculated values and the relatively smaller shift in resonant frequency. These differences may arise from inaccuracies in the empirically estimated simulation parameters, such as the damping constant (*D*_0_) of the sensing element. Additionally, the viscosity of high-concentration glycerol solutions may exceed the assumed range used in constructing the theoretical model, leading to potential distortions in the results.

By identifying the resonance points as the maximum value points on the frequency scan curve, the resonant frequency and resonant amplitude for each measurement result can be determined (Table 1). Under the experimental conditions at 20 °C, the viscosity and density of the glycerol solutions were referenced from existing research findings [40,41]. Repeated measurements of solutions with varying concentrations revealed that the relative standard deviation of the resonant frequency of the response signal was less than 0.03%, and that of the resonant amplitude was less than 0.5%. These results indicate that the experimental setup exhibits excellent repeatability. Furthermore, the standard deviation of the resonant amplitude decreased with increasing solution concentration, suggesting that the measurement error in amplitude is influenced by the damping environment. Specifically, the standard deviation for repeated experiments of the sensitive element in air was 0.1585 mV, which is significantly higher than that for measurements in solution. This finding reflects that the amplitude measurement error is smaller in a high-damping environment, where the mechanical vibration of the sensitive element is more stable. However, the resonant frequency results did not show a similar trend, indicating that the mechanical vibration of the sensitive element is a significant source of measurement error. In a high-damping environment, the amplitude of the sensitive element is reduced, and its vibration is more stable, thereby minimizing the impact of mechanical vibrations on the measurement.

**Figure 4:**
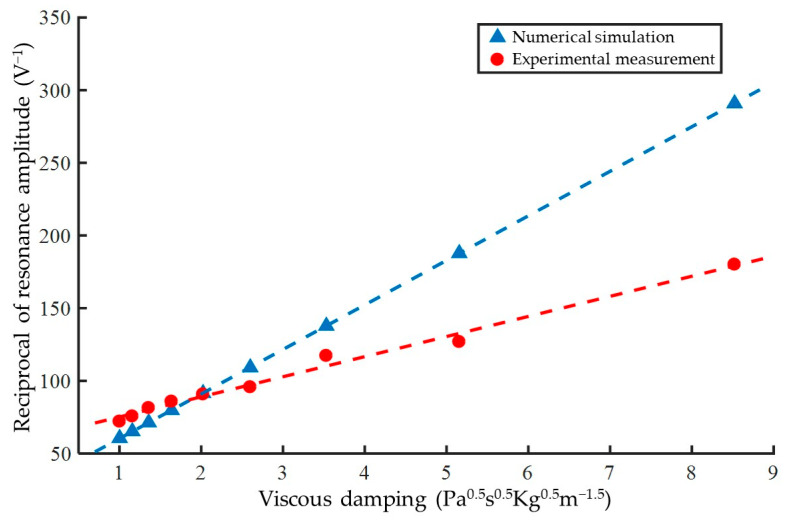
Linearity analysis of resonant voltage amplitude under varying viscosity damping conditions.

Due to the approximations and simplifications introduced during the analytical derivation of Equation (13), numerical simulations based on Equation (10) and experimental measurements were employed for linear fitting to further validate the linear relationship between the reciprocal of the voltage amplitude at the resonance point of the sensor’s response signal and $\sqrt{\eta_{L}\rho_{L}}$ of the measured liquid. A first-degree polynomial was used as the fitting function, and the least squares fitting results are shown in Figure 4. It should be noted that, due to the relatively small standard deviation compared to the mean of the measurement results, error bars are difficult to annotate. Therefore, only the mean values and fitting curves are displayed in the figure, with detailed numerical values provided in Table 1. The coefficient of determination (*R*^2^) for the numerical simulation fitting approaches 1 within the computational accuracy range, indicating that the simplifications in the derivation of Equation (13) did not introduce excessive errors and are reasonable as model conclusions. The *R*^2^ value for the experimental measurement fitting is 0.9886, suggesting that the linear relationship described by Equation (13) is generally consistent with the experimental results. When analyzing the reciprocal of the response voltage as the direct measurement result, the relative standard deviations for each solution are around 0.5%, with no clear pattern observed. It should be clarified that numerical differences between the numerical simulations and experimental measurements are expected due to simplified assumptions in the model and potential errors in simulation parameters. Additionally, manufacturing variations in the sensitive elements introduce some degree of scatter in the measurement results. However, all results conform to a linear relationship and can be corrected through experimental calibration. Therefore, the model supports a magnetoelastic viscosity measurement method based on amplitude analysis. Based on the above measurement and fitting results, and using the response of the sensitive element in air as the blank value, the sensitivity of the magnetoelastic sensing device is calculated to be 13.83 V^−1^/Pa^0.5^s^0.5^Kg^0.5^m^−1.5^, with a detection limit of 0.0817 Pa^0.5^s^0.5^Kg^0.5^m^−1.5^.

### 3.2. Quantitative Assessment of Viscosity Variations During Coagulation

During the coagulation process, fibrinogen is converted into fibrin polymers, leading to a decrease in the fluidity of blood samples and an increase in viscosity. Therefore, real-time magnetoelastic viscosity measurements are performed on blood samples to achieve quantitative monitoring of the coagulation process. Since the overall density of blood samples remains relatively constant during coagulation, the viscous damping value, $\sqrt{\eta_{L}\rho_{L}}$, can be considered equivalent to viscosity. For simplicity, the viscous damping value is used in place of viscosity for subsequent calculations and analyses. The results of the coagulation measurements are analyzed based on the amplitude of the response signal.

**Figure 5:**
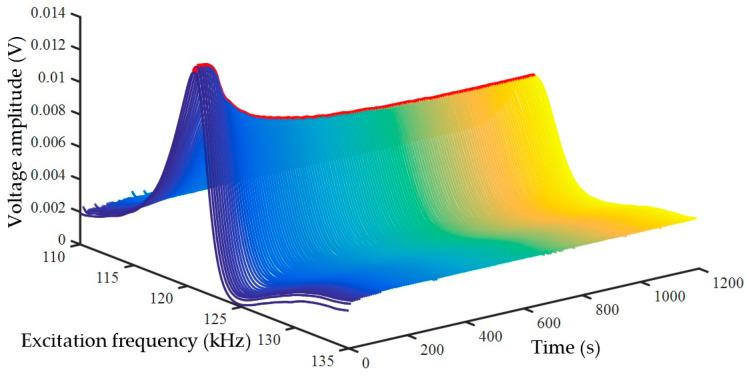
Frequency scanning response during coagulation: the red point indicates the maximum point of the corresponding response curve (the data were randomly selected from repeated experiments).

Given that the sensitive elements in the experiment are not reused, error correction for these elements is necessary during the data processing stage. For illustrative purposes, a random measurement result (Figure 5) from the experiment (the first measurement result of the No. 2 quality control sample) is selected to demonstrate the data processing procedure and viscosity calculation. The coagulation measurement is performed continuously, resulting in a series of response curves obtained from repeated frequency sweeps. To better visualize the temporal progression, a time coordinate is added. As the coagulation reaction progresses, the response curves evolve continuously, with the voltage amplitude at the resonance point decreasing over time. The line connecting the resonance points marked by red dots in the figure actually forms a curve that reflects the coagulation process.

**Figure 6:**
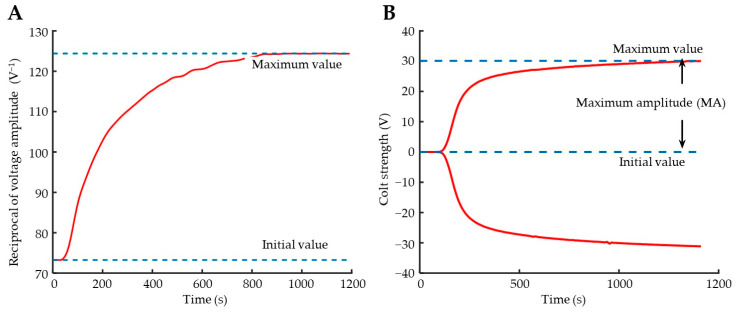
Coagulation profiles derived from example results. (**A**) Change curve of the reciprocal of the magnetoelastic sensor response amplitude (the results were calculated from the data in Figure 5); (**B**) TEG trace obtained simultaneously using the TEG 5000 hemostasis analyzer.

According to Equation (13), based on the detection results in Figure 5, a plot of the reciprocal of the resonance point amplitude over time can be generated to analyze the changes in viscous damping during coagulation (Figure 6A). Upon initiation of the coagulation reaction, the curve rises continuously and remains at its maximum until the reaction is complete.

For reference, simultaneous measurements were conducted using a TEG analyzer, with the results shown in Figure 6B. Based on the detection principle of TEG, the upper and lower curves in the figure are nearly mirror-image symmetric; hence, typically only the upper part of the curve is analyzed. Comparing the curves in Figure 6A,B, it is evident that they share similarities and both reflect the changing characteristics of the coagulation reaction at different stages. These stages include a brief baseline period that appears at the initial activation stage of the coagulation process; an acceleration period where the cascade reaction occurs after the full activation of various coagulation factors; a final stage where fibrinogen completes coagulation, causing the reaction rate to gradually slow down to a stable plateau phase. Compared with the curve in Figure 6B, that in Figure 6A has some noise and fluctuations. This is because the magnetoelastic measurement prototype in this paper cannot match the precision and stability of the mature TEG analyzer. These noises did not significantly affect the subsequent analysis of the coagulation process and relevant parameter calculations, and can be further reduced through future device optimization. Therefore, it can be concluded that magnetoelastic viscosity measurement can qualitatively reflect the coagulation process.

To conduct quantitative analysis, the reciprocal of the measured response amplitude at the resonance point is used to calculate and determine the viscosity changes during the coagulation process. According to Equation (13), the reciprocal of the response amplitude is linearly related to $\sqrt{\eta_{L}\rho_{L}}$. Since the overall density of the sample does not significantly change during coagulation, $\sqrt{\eta_{L}\rho_{L}}$ can be directly used in place of viscosity for subsequent calculations and analyses.

Under fixed detection conditions, the coefficients of the linear term and the constant term in Equation (13) can be considered as constants that are not affected by the measured quantity. However, due to the use of different sensing elements in each measurement, there are certain errors among them, necessitating correction of each measurement result. Furthermore, because the linear term coefficient and constant term involve many physical quantities that may affect the errors, fitting and calculating all these physical quantities is complicated. Therefore, it is convenient to treat the linear term coefficient and constant term as two unknowns, which can be determined through measurements under two specific conditions. The first condition is the pre-measured sensor response in air, where there is no liquid environment. The reciprocal of the response voltage in this case represents the value of the constant term. The second condition is the initial response at the beginning of the coagulation measurement, when the viscosity of the measured sample has not yet changed. Since the viscosity and density of the quality control samples and simulated plasma samples used in the experiment are not significantly different from those of pure water, for the sake of convenience in calculation, the initial viscosity–density product of all samples is simplified and defined as 1. Then, based on the measured initial response and the constant term, the value of the linear term coefficient can be determined. In summary, let *y*_0_ represent the reciprocal of the response amplitude of the sensing element in air, and *y*_1_ represent the reciprocal of the initial response amplitude. For any point on the measured sensor response curve with a reciprocal amplitude *y*, its corresponding viscous damping value *x* can be expressed as:

(14) $$x=\frac{y-y_{0}}{y_{1}-y_{0}}$$

With the assistance of Equation (14), it becomes relatively straightforward to correct for errors among different sensing elements and perform calculations of the viscous damping values. In coagulation monitoring experiments, the measurement results for each type of quality control sample and simulated plasma sample are processed according to the aforementioned procedure. This method enables the magnetoelastic sensor to achieve real-time, quantitative monitoring of viscosity changes during the coagulation reaction, within an acceptable error range.

### 3.3. Comparison with the TEG Analyzer

Thromboelastography (TEG) is a widely used clinical test that analyzes changes in the viscoelastic properties of blood samples during coagulation. Its principle shares some similarities with magnetoelastic sensor-based coagulation monitoring through viscosity measurements. To further evaluate the effectiveness of the magnetoelastic viscosity measurement method based on amplitude analysis for coagulation monitoring, the magnetoelastic measurement results were compared with those obtained from a TEG analyzer. TEG measurement results include a trace proportional to the clot strength of the blood sample, as well as several parameters extracted from this trace. These parameters include: R-time, which reflects the time from the start of measurement to the initial fibrin formation; K-time, which describes the duration of clot formation; α-angle, which indicates the rate of clot formation; and maximum amplitude (MA), which reflects the ultimate strength of the fibrin clot. Among these, the MA is directly related to the viscosity of the tested blood sample and strongly correlates with the $\sqrt{\eta_{L}\rho_{L}}$ obtained from magnetoelastic measurements. Therefore, a comparative analysis was conducted between magnetoelastic measurements and the TEG standard method, based on the MA from TEG and the maximum viscous damping value from magnetoelastic measurements.

**Figure 7:**
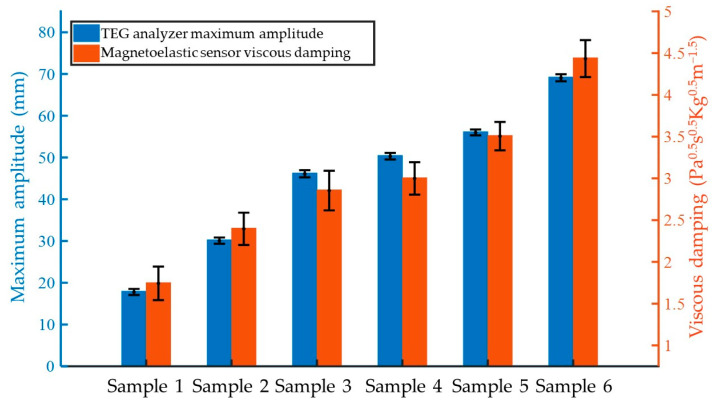
Comparison of quality control sample results: maximum amplitude (MA) from TEG analyzer vs. maximum viscous damping value from magnetoelastic measurement.

The detection results of six quality control samples were compared. The MA values were directly provided by the TEG analyzer, while the maximum viscous damping values were calculated by substituting the maximum value of the magnetoelastic sensor’s response curve into Equation (14). Both sets of results were merged and displayed on the same coordinate axis with appropriate scaling, as shown in Figure 7. The quality control samples had undergone rigorous calibration and were stable in nature. Samples with higher numbers contained relatively more fibrinogen, thus forming stronger clots after coagulation. As seen in Figure 7, from Sample 1 to Sample 6, both the MA measured by the TEG analyzer and the viscous damping value measured by the magnetoelastic sensor gradually increased, indicating a clear correlation between the two. This suggests that using a magnetoelastic sensor for coagulation monitoring can effectively reflect the coagulation characteristics of the tested blood samples.

To further analyze measurement accuracy, the measurement errors of both methods remained relatively consistent across the six quality control samples. However, the accuracy of magnetoelastic measurements was clearly not yet on par with that of the TEG analyzer. Given that the results from the two methods had different dimensions, the relative root mean square error (RRMSE) was employed for quantitative comparison. After calculating the RRMSE for all measurement results, the RRMSE of the MA measured by the TEG analyzer was found to be 1.62%, while the RRMSE of the viscous damping value measured by the magnetoelastic sensing device in this study was 6.57%. In contrast, the relative standard deviation of viscosity measurements for glycerol solutions using the magnetoelastic sensing device was no more than 0.5%. The significantly higher error in the magnetoelastic coagulation monitoring results, compared to glycerol viscosity measurements, can be attributed to two main factors. Firstly, the sensitive elements were not reused during coagulation measurements, and despite corrections, some errors remained. Secondly, the coagulation process is a complex dynamic phenomenon that is more susceptible to external factors compared to static measurements. Additionally, the magnetoelastic sensing device used in this study was not fully optimized for coagulation tests. Unlike the TEG analyzer, it lacked structural designs for sample introduction, mixing, and fixation, which led to more errors being introduced during manual operations. These limitations highlight areas for future improvement to enhance its accuracy and reliability for coagulation monitoring.

**Figure 8:**
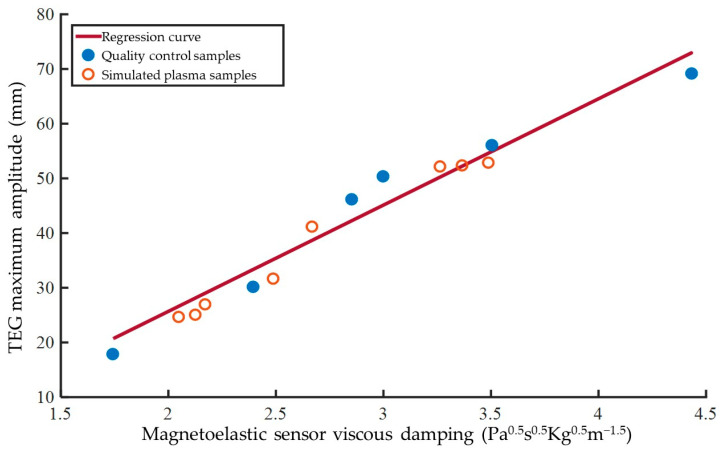
Linear regression analysis and validation between maximum amplitude and maximum viscous damping value.

To further analyze the correlation between the MA and the maximum viscous damping value, a univariate linear regression analysis was conducted on the mean measurement results of six quality control samples. The maximum viscous damping value was used as the independent variable, while MA was treated as the dependent variable. As shown in Figure 8, the six blue solid dots on the graph represent the measurement results of the quality control samples. The horizontal and vertical coordinates of each data point correspond to the mean value of the maximum viscous damping value and the mean value of MA for that sample, respectively. The errors of the measurement results have been previously indicated in Figure 7, and for the sake of clarity, error bars are omitted in this figure. A linear fit was performed on these six points using the least squares method, and the resulting best-fit curve represents the regression curve (the red straight line in Figure 8). The regression equation for this curve is:

(15) $$MA=19.43\cdot \sqrt{\eta_{L}\rho_{L}}-13.19$$

The coefficient of determination (*R*^2^) for this regression equation is calculated to be 0.9546, indicating an approximate first-order linear relationship between the maximum viscous damping value and MA. Additionally, Equation (14) can be reasonably used to convert the maximum viscous damping value to MA. If we consider the mean value of the TEG analyzer measurements as the true value and the MA converted from the mean value of the maximum viscous damping value as the predicted value for error calculation, the RRMSE of the prediction results for this regression model is found to be 7.47%. This error is comparable to the error level of the magnetoelastic measurement of the maximum viscous damping value, suggesting that the error of this conversion is within a reasonable range.

Further validation of the regression model was conducted through comparative measurements of simulated plasma samples. Eight simulated plasma samples were experimentally measured, with the maximum viscous damping value and the corresponding MA serving as the horizontal and vertical coordinates, respectively. These data points were plotted in Figure 8 (as orange hollow dots). Due to the relatively poor stability of simulated plasma, repeated measurements of the same sample were not performed. As shown in Figure 8, the data points for simulated plasma do not deviate significantly from the regression curve. When recalculating the coefficient of determination *R*^2^ for the regression equation using the simulated plasma data, the obtained value was 0.9615, which shows no significant difference compared to that of the quality control samples. The regression equation can be used to convert the maximum viscous damping value to MA, with the RRMSE for the conversion of simulated plasma data being 5.84%. This error level is comparable to that of the magnetoelastic measurement of the maximum viscous damping value and has not increased compared to the quality control samples. Based on these results, it can be concluded that the maximum viscous damping value obtained through magnetoelastic measurements is comparable to the MA parameter of TEG and can reasonably reflect the final clot strength of the tested blood samples. Furthermore, it can be converted to MA within an acceptable error range using Equation (14).

**Figure 9:**
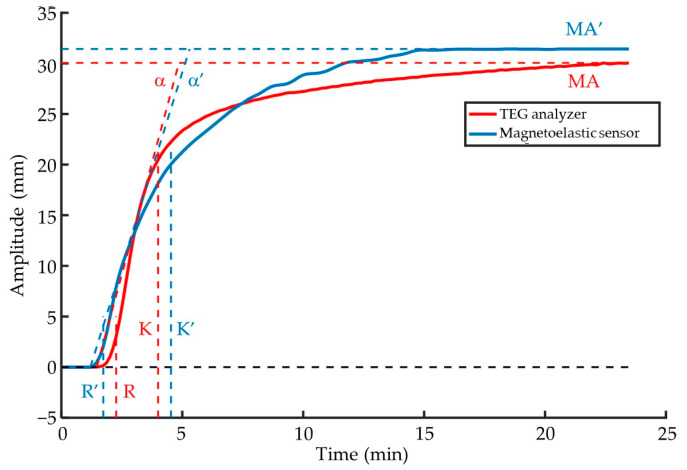
Comparison of the TEG trace converted from magnetoelastic measurement results with that measured by the TEG analyzer (showing only the top half), and extraction of R, K, α-angle, and MA parameters.

Based on the above analysis, the conversion from the maximum viscous damping value to MA using Equation (14) enables the mapping of changes in the viscous damping value of samples during coagulation into changes in the amplitude of the TEG trace. This allows for the transformation of any point on the response curve of the magnetoelastic sensor, ultimately converting the resonant amplitude curve of the magnetoelastic sensor into a TEG trace. For example, the magnetoelastic measurement curve shown in Figure 6A is converted into a TEG trace, represented by the blue curve in Figure 9. For comparison, the TEG trace measured by the TEG analyzer (originally shown in Figure 6B) is plotted as the red curve in Figure 9. It can be observed that the basic shapes of the two curves are similar, although some differences remain. Considering the TEG trace measured by the TEG analyzer as the standard value and the converted curve from magnetoelastic measurements as the predicted value, the RRMSE of this conversion is 7.45%, and the coefficient of determination *R*^2^ is 0.9552. These results suggest that this conversion method is feasible.

After converting the resonant amplitude curve of the magnetoelastic sensor into a TEG trace, other common TEG parameters mentioned earlier can be further obtained by referring to the standard definitions of TEG, as shown in Figure 9. The parameters extracted from the TEG trace measured by the TEG analyzer are labeled as R, K, α-angle, and MA, while those extracted from the converted TEG trace are labeled as R′, K′, α-angle’, and MA′. Following this procedure, curve conversion and parameter extraction were performed for the five repeated experimental results of the quality control samples corresponding to the examples in the figure. The TEG analyzer measured an R-time of 2.2 ± 0.2 min, K-time of 1.9 ± 0.1 min, α-angle of 82.7 ± 1.0°, and MA of 30.1 ± 0.7 mm. In contrast, the magnetoelastic sensor measured an R-time of 1.9 ± 0.3 min, K-time of 2.3 ± 0.6 min, α-angle of 77.4 ± 8.9°, and MA of 32.3 ± 2.2 mm. Taking the mean values measured by the TEG analyzer as the standard values, the relative standard deviations of the four parameters measured by the magnetoelastic sensor were calculated to be 13.6% for R-time, 21.0% for K-time, 6.4% for α-angle, and 7.3% for MA. Among these results, the errors for R-time and K-time are relatively large. Possible reasons include timing errors due to manual sample loading in magnetoelastic testing and inadequate mixing of the tested blood samples with the activator, which may lead to differences in the rate of coagulation reactions and affect related parameters. The TEG analyzer’s experimental results show a smaller K-time mean and larger α-angle mean than the magnetoelastic results, indicating faster clot formation in TEG measurements. This is also confirmed by the coagulation curve in Figure 9. The possible reason is that in magnetoelastic detection, the small test tube used as the sample pool has limited space, making it difficult to fully mix the sample with the activator during sampling, thus affecting the coagulation reaction rate. In contrast, the TEG analyzer uses a wide-mouthed sample cup, allowing for better mixing during sampling. Additionally, the continuous macroscopic mechanical motion during TEG measurement helps accelerate mixing. Moreover, it is unclear whether the contact of the sensitive element surface affects the coagulation process in magnetoelastic detection, and its impact on the measurement cannot be ruled out. Furthermore, manual sampling in the current experiment leads to significant errors in the R and K times of the magnetic elastic detection, and further optimization of the device is needed to reduce these errors. Based on the comprehensive analysis of the results, the magnetoelastic sensor can achieve real-time, quantitative monitoring of viscosity changes during coagulation and can be converted into standard TEG results within a certain error range. This indicates that the magnetoelastic viscosity sensing method based on amplitude analysis is highly feasible for coagulation monitoring.

## 4. Conclusions

This paper introduces a novel magnetoelastic viscosity sensing method based on amplitude analysis, achieved through the reconstruction of a theoretical model. The method is applied to real-time, quantitative monitoring of the coagulation process. Using the sensing device developed based on this approach, the viscosity variation curve during coagulation can be measured quantitatively in real time. With the aid of a regression model, the viscosity results can be further converted into a thromboelastograph (TEG) trace, thereby reflecting the dynamic process of coagulation. Compared to conventional magnetoelastic sensing techniques, this method offers a simpler device design and lower cost, enabling convenient and quantitative detection. It holds significant potential for application in coagulation monitoring.

## Figures and Tables

**Table 1:** Resonant frequency and voltage amplitude results of glycerol solution.

Concentration (wt%)	Viscosity *η_L_* (10^−3^ Pa·s)	$\mathrm{Density}\;\rho_{L}(\mathrm{kg}/m^{3})$	Resonant Frequency (Hz)	Resonant Amplitude (mV)
0	1.01	998.23	120,898.3 ± 9.1	13.8837 ± 0.0350
10	1.31	1022.10	120,643.3 ± 17.3	13.2270 ± 0.0561
20	1.76	1046.90	120,308.3 ± 23.1	12.2919 ± 0.0563
30	2.50	1072.70	119,950.0 ± 13.1	11.6586 ± 0.0227
40	3.72	1099.30	119,441.7 ± 19.0	11.0223 ± 0.0415
50	6.00	1126.30	118,940.0 ± 24.2	10.4544 ± 0.0221
60	10.80	1153.80	118,470.0 ± 28.2	8.5304 ± 0.0167
70	22.50	1181.25	117,686.4 ± 23.4	7.8858 ± 0.0194
80	60.10	1208.50	116,390.5 ± 30.1	5.5546 ± 0.0029

## Data Availability

Data are contained within the article.
